# Determining the acceptability of testing contacts of confirmed COVID-19 cases to improve secondary case ascertainment

**DOI:** 10.1093/pubmed/fdab079

**Published:** 2021-03-30

**Authors:** E Marchant, D Ready, G Wimbury, R Smithson, A Charlett, I Oliver

**Affiliations:** 1 UK Field Epidemiology Training Programme, Global Public Health Division, Public Health England, London NW9 5EQ, UK; 2 Field Services, South West England, Public Health England, Bristol BS1 6EH, UK; 3 NIHR Health Protection Research Unit in Behavioural Science and Evaluation, University of Bristol, BS8 1QU, UK; 4 NHS Test & Trace Programme, Department of Health and Social Care, London SW1H 0TL, UK; 5 Statistics, Modelling, and Economics, National Infection Service, Public Health England, London, NW9 5EQ, UK; 6 National Infection Service, Public Health England, London SE1 8UG, UK

**Keywords:** behaviour, communicable diseases, health protection

## Abstract

**Background:**

UK asymptomatic contacts of confirmed COVID-19 cases are not routinely tested for SARS-CoV-2. Testing contacts may improve case ascertainment and reduce onward transmission. This study investigated the acceptability of SARS-CoV-2 testing among contacts of confirmed cases as an integral part of the contact-tracing process.

**Methods:**

A cross-sectional descriptive survey of case contacts was conducted in the UK. All contacts who completed a telephone call with the NHS Test and Trace Agile Lighthouse team were eligible for inclusion and were offered a molecular test. Consenting participants were sent a self-swab kit.

**Results:**

Of the 1523 individuals contacted, 602 (39.5%) accepted the test offer. Of the 240 (39.9%) samples returned for testing, 16.3% tested polymerase chain reaction-positive for SARS-CoV-2.

Most individuals who declined with a reason (638/905; 70.5%) reported they had already taken or booked a SARS-CoV-2 test, or were part of a testing programme. Matched laboratory records confirmed 73.1% of those who declined were tested by another route.

**Conclusions:**

Most case contacts were tested, either through arranging a test by themselves or by accepting the study offer. Results demonstrate high acceptability, with substantial test positivity, indicating that there is public health benefit in offering tests to contacts as a routine part of the contact-tracing process.

## Introduction

On 11 March 2020, the World Health Organization declared the outbreak of COVID-19 a pandemic following confirmation of global spread.^1^ As of 22 January 2021 (10.00 am CET), over 94 million cases have been diagnosed globally, with over 2 million fatalities. In the 14 days till 22 January, over 10 million cases were reported.^2^ In the UK, there have been 3 959 784 laboratory-confirmed cases as of 08 February 2021 (9.00 am GMT) and 112 798 deaths within 28 days of a positive test. ^3^

Pivotal to the control of COVID-19 is case ascertainment and contact-tracing. NHS Test and Trace (NHSTT) was launched in the UK on 28 May 2020 to ensure timely access to SARS-CoV-2 testing for symptomatic individuals and to trace the close contacts of all confirmed COVID-19 cases.^4^ At present, contacts of confirmed cases of COVID-19 are not routinely tested for SARS-CoV-2 infection, though they can access SARS-CoV-2 testing if they develop cardinal symptoms of COVID-19. Routine testing of contacts of cases could improve case ascertainment through identification of asymptomatic, paucisymptomatic and pre-symptomatic cases. In turn, this could be used to support a strategy to reduce transmission of COVID-19 as part of the COVID-19 response.

This study sought to investigate the acceptability of testing the contacts of confirmed cases of COVID-19 as part of routine contact-tracing and explored barriers to testing among this population. The primary objectives were to determine, among contacts who were offered a SARS-CoV-2 test:

- the proportion who stated this was acceptable and would consent to testing, having been appropriately briefed of the implications;
- the proportion who returned a completed swab following despatch and
- the proportion who completed a home swab which yielded a valid PCR result.

Secondary objectives were to:

- collect reasons for declining testing when offered;
- describe the demographics of those agreeing and declining to participate;
- collect details of symptoms presenting in the 14 days before self-sampling;
- describe the demographics of those successfully completing the pilot and
- describe the interval between decision to test and the result being available in the laboratory and other relevant intervals in the specimen journey.

## Methods

A cross-sectional descriptive survey of a sample of contacts of confirmed cases of COVID-19 in UK was conducted.

Individuals were recruited to the study by a ring-fenced team of 30 NHSTT call handlers working in the Agile Lighthouse team. All contacts of confirmed cases, identified through routine contact-tracing, who completed the telephone call by this team were eligible for inclusion. Contacts, or their parents/guardians, were asked if they would be happy to receive a self-swab test for SARS-CoV-2; testing was serially offered to all individuals who were contacted by the Agile Lighthouse team each day. All recruited individuals were sent a self-swab kit plus instructions in the post. Recruitment took place over a 3.5-week period between 24 September and 19 October 2020. Recruitment ended either when 800 individuals had accepted the offer of a test kit, or at the end of the predefined period.

Recruitment for the study took place Monday to Friday. The NHSTT team provided a list of individuals to Public Health England (PHE) each morning following recruitment, and this list was used to send out postal kits to participants. Trackable self-sample kits were posted to participants by PHE using Royal Mail delivery between 25 September 2020 and 21 October 2020.

Samples were returned to PHE by an approved, trackable Royal Mail postal route for SARS-CoV-2 specimens. Swabs were received in the PHE lab from 29 September 2020 to 02 November 2020 and were tested by reverse transcriptase polymerase chain reaction (RT-PCR) that was designed and evaluated for the detection of SARS-CoV-2 in clinical respiratory samples, using the ABI QuantStudio 7 Flex real-time PCR system. The assay specifically detected SARS-CoV-2 in the Orf1ab assay target and Sarbecoviruses including SARS CoV-2 in the E gene target. Participants in this study were notified of their result by text message. Individuals with a positive result were given advice through the routine NHSTT process.

### Sample size

The study aimed to recruit 800 contacts for SARS-CoV-2 testing based on the number of eligible contacts expected over a 2-week recruitment period. A sample size of 600 subjects had sufficient power to detect a difference in proportions of 0.15 between the two groups regardless of the proportion in the comparison group or the ratio of the group sizes, unless this was very extreme.

### Data collection

We collected information on the acceptance and reasons for declining. Demographic information was obtained from the NHSTT records. NHS numbers were obtained where possible from the NHS Demographic Batch Tracing Service (DBS). This data were linked to Hospital Episode Statistics to obtain information of ethnicity from the NHS Spine.

The self-sample swab kit contained a laboratory request form which participants were asked to complete and return with their completed swab. This form collected demographic information and symptomatology. The laboratory request forms were uploaded to the PHE Laboratory Information System records along with SARS-CoV-2 test results.

Deterministic linkage was used to link information on participants to the PHE Second Generation Surveillance System (SGSS), using NHS number on 02 November 2020, to identify if individuals from this study were tested via another route during the study period (24 September–02 November 2020). A second linkage was carried out on 16 November 2020, based on the person’s name, date of birth and/or postcode. For individuals who did not return a test kit, laboratory results were obtained for samples either within the study period (24 September–02 November 2020) or within 90 days prior to the first day of study recruitment (26 June 2020). This period was chosen to exclude tests taken due to previous infections since viral ribonucleic acid (RNA) PCR positivity can occur for up to 3 months in immunocompetent individuals. The notable result was taken to be the earliest positive result in the period, and if no positive result then the first negative, both based on sample date.

### Data analysis

Data were analysed using Stata 15.1. Chi,^2^ and rank sum tests were used to determine the difference in proportion and distribution between the two study groups, respectively. Ninety-five percent confidence intervals (CIs) for proportions have been calculated using the Clopper-Pearson exact method, and for medians, using the method of Mood and Graybill.

### Ethical committee clearance

Research governance approval for this study was granted by PHE Research Ethics and Governance Group on 23 September 2020 (reference NR0235).

## Results

### Acceptability of testing

A total of 1,523 contacts of confirmed cases of COVID-19 were asked if they would find having a test for SARS-CoV-2 acceptable and would consent to testing. Of these, 39.5% (602/1,523) consented to testing.

Basic demographic information was available for 86.0% (518/602) of individuals who found testing acceptable and for 66.7% (614/921) who found testing unacceptable. Ethnicity information was available for 62.6% (377/602) of individuals who found testing acceptable and for 50.3% (463/921) who found testing unacceptable.

Those who accepted the offer of testing were more likely to be male (50.0% versus 46.2%, *P* = 0.01) and younger (median age of 25 versus 30, *P* < 0.01) as compared to those who declined the offer of testing. Among all participants, 84.9% were of white ethnicity, and the majority were in the Midlands, North West or North East Yorkshire and Humber regions of the UK. There were no important differences identified in the ethnicity or geographical distribution between those who accepted and those who declined PCR testing (Table 1).

**Table 1:** Demographics of contacts of confirmed cases of COVID-19 who find testing for SARS-CoV-2 acceptable and unacceptable (*n* = 1,213)

	*Offer accepted* *(n = 567)*		*Offer not accepted* *(n = 646)*		**P*-value*	*Test returned (*n* = 226)*	
	*Proportion*	*95% CI*	*Proportion*	*95% CI*		*Proportion*	*95% CI*
Sex^a^					0.01		
Male	50.0% (284/567)	45.9–54.3%	46.2% (295/638)	42.3–50.2%		51.3% (116/226)	44.6–58.0%
Female	50.0% (283/567)	45.7–54.1%	53.8% (343/638)	49.8–57.7%		48.7% (110/226)	42.0–55.4%
Age^b^							
Median	25	23–27	30	27.6–32	<0.01	28	24–32
Interquartile range	16–41		19–49			15–46	
Range	0–83		0–101			0–83	
Geography^c^					0.15		
East of England	5.4% (28/518)	3.6–7.7%	4.6% (28/614)	3.1–6.5%		3.3% (7/209)	1.4–6.8%
London	9.8% (51/518)	7.4–12.7%	7.7% (47/614)	5.7–10.0%		7.2% (15/209)	4.1–11.6%
Midlands	17.2% (89/518)	14.0–20.7%	19.4% (119/614)	16.3–22.7%		15.8% (33/209)	11.1–21.5%
North East, Yorkshire and Humber	29.0% (150/518)	25.1–33.1%	27.5% (169/614)	24.0–31.2%		32.1% (67/209)	25.8–38.8%
North West	24.1% (125/518)	20.5–28.1%	27.5% (169/614)	24.0–31.2%		25.8% (54/209)	20.0–32.3%
South East	8.5% (44/518)	6.2–11.2%	8.0% (49/614)	6.0–10.4%		9.6% (20/209)	5.9–14.4%
South West	6.0% (31/518)	4.1–8.4%	5.4% (33/614)	3.7–7.5%		6.2% (13/209)	3.4–10.4%
Ethnicity^d^					0.45		
Asian	8.2% (31/377)	5.7–11.5%	10.8% (50/463)	8.1–14.0%		4.7% (8/172)	2.0–9.0%
Black	2.4% (9/377)	11.0–4.5%	1.7% (8/463)	0.7–3.4%		0.6% (1/172)	0.0–0.3%
Mixed	1.6% (6/377)	0.6–3.4%	1.1% (5/463)	0.4–2.5%		1.2% (2/172)	0.0–0.4%
White	84.9% (320/377)	80.9–88.3%	84.9% (393/463)	81.3–88.0%		80.8% (139/172)	7.4–8.6%
Other	2.9% (11/377)	1.5–5.2%	1.5% (7/463)	6.1–3.1%		2.3% (4/172)	0.0–0.6%

^a^Data completeness for sex: *n* = 567 testing acceptable, *n* = 638 testing unacceptable and *n* = 226 test returned.

^b^Data completeness for age: *n* = 526 testing acceptable, *n* = 646 testing unacceptable and *n* = 210 test returned.

^c^Data completeness for geography: *n* = 518 testing acceptable, *n* = 614 testing unacceptable and *n* = 209 test returned.

^d^Data completeness for ethnicity: *n* = 377 testing acceptable, *n* = 463 testing unacceptable and *n* = 172 test returned.

### Test return

Overall, 240 self-collected swabs were returned to the PHE laboratory for SARS-CoV-2 testing, a return rate of 39.9% (240/602). Of those who returned a completed swab and for whom demographic information was available, 51.3% (116/226) were male with a median age of 28 and 80.8% (139/172) were of white ethnicity (Table 1).

Four swabs were returned from individuals not recruited into this study. These may have been undisclosed contacts or other household members. Three of these samples were positive for SARS-CoV-2 RNA.

### Test positivity

All 240 samples returned yielded a valid result, with 16.3% (39/240) of the samples tested being SARS-CoV-2 positive. The median cycle threshold (ct) value for the PCR-positive samples was 28.6 (range: 13.6–35.6) for the ORF1ab gene and 30.0 (range: 12.9–35.9) for the E gene target. One PCR result was positive for the E gene target and negative for the ORF1ab gene.

Those testing positive were more likely to be male (60.6% compared to 50.5% among individuals testing negative), however, these differences were not statistically significant (*P* = 0.58 and *P* = 0.87, respectively). Of the individuals who tested positive, 51.3% (20/39) reported symptoms in the 14 days before collection of a swab compared with 14.9% (29/195) among those testing negative, and this difference was statistically significant (*P* < 0.01). Symptomatic individuals would have been able to access a test via an existing route, so theoretically, these individuals could have been tested and identified by existing pathways. There was no observable difference in the median age between those testing positive and those testing negative (Table 2).

**Table 2:** Demographics of contacts of confirmed cases of COVID-19 who returned a completed test for SARS-CoV-2 (*n* = 240) as at 3 November 2020

	*Test positive (*n* = 39)*		*Test negative (*n* = 201)*		
	*Proportion*	*95% CI*	*Proportion*	*95% CI*	**P*-value*
Sex^a^					
Male	60.6% (20/33)	42.1–77.1%	50.5% (96/190)	43.2–57.8%	0.58
Age^b^					
Median	29	15.7–45	28	24–32	0.87
Interquartile range	11–54		15–44		
Range	0–82		1–83		
Ethnicity^c^					
White	87.5% (21/24)	67.6–97.3%	79.7% (118/148)	72.3–85.9%	0.83
Symptoms^d^					
Yes	51.3% (20/39)	34.8–67.6%	14.9% (29/195)	10.2–20.7%	<0.01

^a^Data completeness for sex: *n* = 33 test positive and *n* = 190 testing negative.

^b^Data completeness for age: *n* = 33 test positive and *n* = 177 testing negative.

^c^Data completeness for ethnicity: *n* = 24 test positive and *n* = 148 testing negative, self-reported ethnicity on lab request form.

^d^Data completeness for symptoms: *n* = 39 test positive and *n* = 195 testing negative.

### Timeliness

The median time taken from notification and recruitment by the NHSTT team to an individual taking the swab was 5 days (range: 1–16 days). The median time taken from the collection of the sample to receipt in the laboratory using the approved Royal Mail delivery was 1 day (range: 0–6 days) and to process the samples in the laboratory to deliver a validated result was 1 day. Therefore, the median length of time taken from the recruitment to the notification of a positive result to NHSTT was 7 days (range: 4–20 days). Results were available for 99.8% of the contacts before the end of their 14-day quarantine period.

### Reasons for declining a test

Information on the reason for declining the offer of testing was collected for 98.3% (905/921) of the contacts who declined. A total of 638/905 (70.5%) of these individuals stated that they; 1) had already had a test by the time of the call (408/905; 45.1%), 2) they had already booked a test (218/905; 24.1%), or 3) were part of a routine testing programme (12/905; 1.33%). There were 117 (12.9%) individuals who advised that they did not want a test and 85 (9.4%) people declined because they were asymptomatic and therefore reported a test was not needed. Others refused the offer because they self-reported having a previous negative SARS-CoV-2 result (8.2%), not wishing to use resources that could be directed to those in more need (0.6%) or being too young (3.0%).

### Validation of testing histories for those not completing sampling via the pilot

**Fig. 1:**
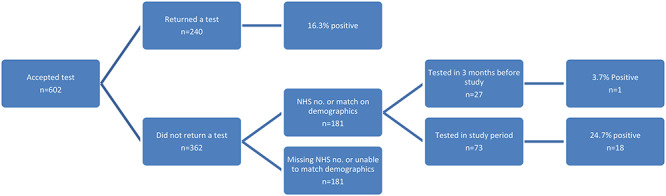
Flow chart of testing among those accepting testing (*n* = 602).

**Fig. 2:**
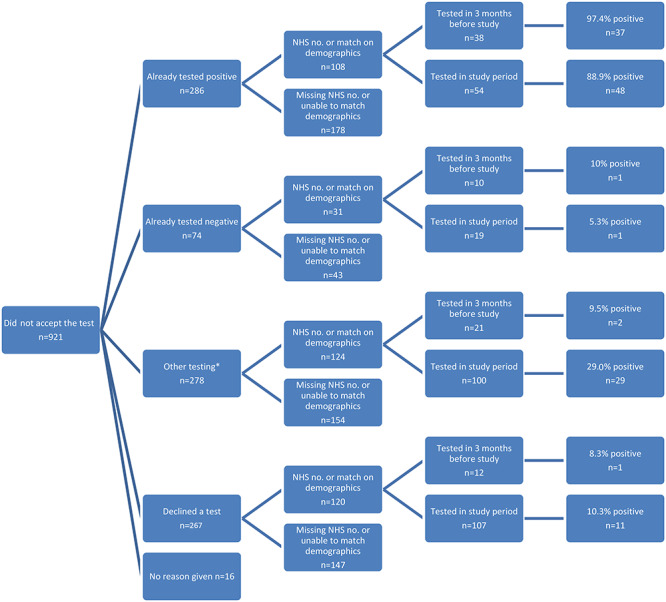
Flow chart of validation of testing histories for those not completing sampling via the pilot (*n* = 921). Asterisk (*) denotes that other testing is a grouping of regular testing, awaiting a test, awaiting a result and no result disclosed.

In total, there were 1,283 people in the study who either did not accept a test offer (*n* = 921) or accepted the offer but did not return the swab provided to them (*n* = 362). After matching on the NHS number, or full name with date of birth or postcode, laboratory records were obtained for 50.0% (181/362) of those who consented to testing but did not return a test kit (Fig. 1) and 41.6% (383/921) of those who did not consent to testing. (Fig. 2).

Of the 181 individuals who accepted testing but did not return a swab and were matched to laboratory records, 40.3% (73/181) had a test within the study period (24 September–02 November 2020). Of these, 24.7% (18/73) were positive for SARS-CoV-2. A further 27 (14.9%) individuals had a test within 3 months of the start of the study period (Fig. 1).

Of the 383 individuals who declined testing and were matched to laboratory records, 73.1% (280/383) had a test within the study period. Overall, 31.8% (89/280) of these results were positive. A further 81 (21.1%) were tested within the 3 months prior to the study start date. (Fig. 2).

## Discussion

### Main findings of this study

A total of 1523 individuals were contacted by NHSTT, and 39.5% (602/1523) accepted the offer of testing and were sent self-swab kits. The majority of those contacted did not accept the offer of a test kit (*n* = 921; 60.5%); however, most of those who provided a reason (638/905; 70.5%) reported that they had already been tested, booked a test or were part of a routine testing programme. Laboratory records confirmed that a high proportion of those who declined the offer of a test kit were tested by another route during the study period.

Self-sample swab kits were posted to 602 participants by PHE for PCR testing for SARS-CoV-2. Only 39.9% (240/602) of the postal test kits were returned; however, this is likely to underestimate compliance with testing as laboratory results identified an additional 73 (40.3%) of 181 individuals with NHS numbers that were tested for SARS-CoV-2 by PCR via an alternative route during the study period. The majority of case contacts identified through the NHSTT programme therefore appear to find testing for contacts of cases acceptable, though many stated that they had already arranged a test themselves by the time they were followed up by contact tracers. It is also possible that contacts chose to keep the swab kits ‘just in case’ they wanted to take a test at a later stage, although this will need to be confirmed.

The positivity rate for individuals who returned a study test kit was 16.3% (39/240). Individuals who tested positive were statistically more likely to report symptoms in the previous 14 days (51.3%) compared with those testing negative (14.9%; *P* < 0.01). Even taking into account the individuals who reported symptoms and would have qualified for pillar 2 testing, the 16.3% positivity rate in returned samples indicates that case finding through routine testing of all contacts of confirmed cases would add significant public health value. This approach may also decrease the time to obtain a test result for individuals who are pre-symptomatic or paucisymptomatic at the time they engage with NHSTT.

The overall positivity rate during the study period for those whose NHS records were retrievable and were tested in the study period was 24.6% (146/593), comprising: 39/240 who returned a study test kit; 18/73 who consented to receive a test kit but were tested by another route and 89/280 who declined to receive a test kit but were tested by another route.

### What is already known on this topic

At present, there are no published studies on the acceptability of testing contacts of confirmed cases of COVID-19 in the UK. Pivotal to the control of COVID-19 is case finding and contact-tracing, and this will only be successful if people find this process acceptable and comply with the offer of testing. Further efforts are needed to reduce the transmission of SARS-CoV-2 and further understanding is needed of the benefits that testing contacts may have on case ascertainment and transmission.

### What this study adds

This study is one of the first looking at the acceptability of testing contacts of confirmed cases of COVID-19 in the UK. While the majority of individual contacts did not accept an offer of a test, this study suggests that the majority of contacts appear to want a test as many had already arranged a test by the time they are followed up by contact tracers. Most of those who gave a reason for declining a test stated that they already had a test, booked a test or were part of a routine testing programme which was validated for many through laboratory records. Therefore, overall testing appears to be acceptable to most contacts of COVID-19 cases. However, there is a potential communication need as 8.1% (74/921) of people who refused the offer of a test stated that this was due to having an existing negative result, which implies that they had not realised that they may subsequently become infectious over the incubation period (14 days) or acquire the infection from another source.

Looking at acceptability and test positivity, this study suggests that there is likely to be significant public health benefit in routinely offering SARS-CoV-2 tests to first-degree contacts as part of the contact-tracing process in order to increase case ascertainment and reduce the time to test result for individuals.

The low return rate of the study (excluding tests by other routes) highlights a difference between a testing programme being acceptable in theory and successful in practice. Further investigation work would be needed to understand the barriers to returning test kits. In addition, low return rates for postal swab kits may impact on the cost-effectiveness of a testing programme, so alternative delivery models should be investigated.

This study was not designed to assess the optimal methodology, timing or frequency of SARS-CoV-2 test delivery for the contacts of confirmed cases. Work is in progress to assess these factors to maximize the benefits of the programme, and these would need to be carefully considered for the design of a success programme.

### Limitations of this study

There are limitations to this study. We did not reach the desired 800 samples for recruitment and kits were not sent to participants at the weekends, which would reduce the timeliness to result. Data quality issues meant that NHS numbers and demographic details were not always available, and it was not possible to access laboratory records for all participants who declined a test or did not return a test kit.

## Funding

This work was funded by Public Health England.

## Data availability

The data underlying this article cannot be shared publicly due to information governance regulations and the need to preserve the confidentiality of individuals who participated in the study.

## Notes

**E. Marchant,** FETP Fellow

**D. Ready,** Consultant in Public Health Infections, National Infection Service

**G. Wimbury,** Agile Lighthouse Lead, DHSC

**R. Smithson,** Agile Lighthouse Team Lead, DHSC

**A. Charlett,** Department Head, Statistics, Modelling and Economics, Data and Analytical Services

**I. Oliver,** Director, National Infection Service
